# Hearing the Voices of Wingless Angels: A Critical Content Analysis of Nurses’ COVID-19 Experiences

**DOI:** 10.3390/ijerph17228484

**Published:** 2020-11-16

**Authors:** Huseyin Arasli, Trude Furunes, Kaveh Jafari, Mehmet Bahri Saydam, Zehra Degirmencioglu

**Affiliations:** 1 Norwegian School of Hotel Management, University of Stavanger, 4036 Stavanger, Norway; trude.furunes@uis.no; 2 Faculty of Tourism, Eastern Mediterranean University, TRNC, Via Mersin 10, Gazimagusa 99628, Turkey; kavehjafari91@gmail.com (K.J.); mehmet.saydam@emu.edu.tr (M.B.S.); 3 English Preparatory School, Cyprus West University, TRNC, Via Mersin 10, Gazimagusa 99450, Turkey; z.degirmencioglu@cwu.edu.tr

**Keywords:** nurses, COVID-19, content analysis, nurses’ perception, Instagram, social media

## Abstract

The world has been affected by an outbreak of the novel coronavirus (COVID-19). Health care workers are among those most at risk of contracting the virus. In the fight against the coronavirus, nurses play a critical role. Still, most social media platforms demonstrate that nurses fear that their health is not being prioritized. The purpose of this study is to investigate nurses’ experiences through analyzing the main themes shared on Instagram by nurses during the COVID-19 pandemic. In contrast with highly structured research, the current paper highlights nurses’ natural language use in describing their experiences during the first months of the outbreak in their workplace. Instagram captions were utilized as a data source. Leximancer was utilized for the content analysis of nurses’ narratives towards their coronavirus experience. We sought to accomplish three research objectives: the first was to identify the main themes in the descriptions of nurses’ experiences shared via their social media, specifically Instagram; then, to determine the relationships among concepts, and finally, to give useful implications based on the findings. The current study uses a qualitative (i.e., narratives) approach to analyze the main components of the nurses’ experiences during the pandemic. The Leximancer software analysis revealed nine major textual themes and the relationships among these themes. In order of the relative importance, the themes were “patients”, “coronavirus”, “exhaustion”, “family”, “hospital”, “personal protective equipment” (PPE), “shift”, “fear”, and “uncertainty”. The results offer practical implications based on the social media information regarding nurses’ overall experiences.

## 1. Introduction

In the early months of 2020, a novel virus, coronavirus (COVID-19), became an international issue [1]. On the 30th of January, the World Health Organization confirmed a global health emergency, and on 11th March, a pandemic. The fast, global spread of COVID-19 caused severe impairments to countries, producing deadly health risks, job losses, and a worsening work–life balance [2]. Consequently, a state of emergency was declared globally due to COVID-19, and efforts are being made to overcome the current situation [3]. This includes nurses taking care of COVID-19 patients at the scenes of COVID-19 contaminations, where they must wear protective equipment for long hours, leaving marks on their faces [4]. However, as COVID-19 has been continuing interminably, health care workers are encountering hardships in carrying out their jobs [5,6,7,8,9].

In every country, nurses are being praised for their efforts, and countless encouraging messages are relayed to medical workers through social media platforms. Nurses play an important role in COVID-19 prevention and response efforts. The nursing profession is a leading profession internationally, with approximately 3.8 million nurses in the United States (US) and over 20 million nurses worldwide.

Nurses assumed the closest contact with COVID-19 patients during this period. Therefore, nurses have undertaken the most important tasks in rescuing critically ill patients and curing infected patients in the COVID-19 period [10]. While this is the case, healthcare institutions stated that they would need more healthcare professionals as the pandemic increases [11,12]. Caring is the core of the nursing profession and is central to their role in developing effective relationships with the individuals they help [13,14,15]. As a trusted health professional group, nurses also play a key role in disease prevention and reducing the spread of the outbreak [10].

Nurses experience mental stress and may feel isolated and abandoned in the face of health fears and burdens from the high-intensity work triggered by this type of public health tragedy [16]. Earlier research has displayed that when healthcare workers are in close contact with patients with transmissible viruses, for example severe acute respiratory syndrome (SARS), Middle East respiratory syndrome coronavirus (MERS-Cov), Ebola, and swine flu (H1N1), they struggle from isolation, nervousness, anxiety, exhaustion, sleep disorders, and other physical and mental health difficulties [16].

Research has revealed that the occurrence of depression, insomnia, and post-traumatic stress among nurses involved in the treatment of SARS patients was 38.5%, 37%, and 33%, respectively [17]. In an investigation on the psychological level of Ebola patients’ nurses, 29% of participants reported loneliness, and 45% received psychological therapy [18]. For the reason that COVID-19 is a novel virus, and the remedial system and culture of different countries differs, more studies are necessary to evaluate the experience of frontline nurses fighting against COVID-19 [16,19]. Presently, the published research has stressed the virus incidence [20], clinical characteristics, diagnosis, and treatment [21]. However, no research has been published on the COVID-19 experiences of nurses, using user-generated content.

Several nurses have shared their experiences via their social media accounts, which was reflected in newspapers. For example, a nurse in the United States (US) used a social media platform to show the poor working settings that might put ill people at jeopardy. “*It is disgusting*”, one nurse wrote. More than 1200 nurses shared a private online document to share their stories of combatting the COVID-19 pandemic on the front lines. In their profiles, they stated that the outbreak had turned US hospitals into “war zones.” They stated that they were frightened to go to work and worried that they will become ill. They complained about the top management of hospitals who seem careless towards their difficulty [22].

Another nurse in Italy shared a picture through her Instagram account saying that “I afraid to go to work” during the pandemic, but she continued to do so since she “honored” and respected her profession. She also added that “I am frightened since the personal protection equipment didn’t provided enough”. She also added that she was mentally exhausted. On her Instagram account, she stated that “the lab coat makes me sweat,” and that, once she is dressed, she is not allowed to “go to the restroom or drink for seven hours” in order to diminish any possible virus spread. Her post was liked over 800,000 times, and followers shared supportive and appreciative comments for all nurses in this post [23]. As seen above, social media is seen as a representative channel to reflect the nurses’ experiences and perceptions. 

Understanding nurses’ experiences is essential to ensure that appropriate support is given to enable labor retention and high-quality clinical practice at a time when society’s health necessities are extraordinary. However, academically, inadequate research has been published regarding the experiences of nurses during a pandemic [16,19]. The recent literature on the experiences of nurses was based on surveys. Additional methods based on the analysis of the online reviews shared by users in online communities, blogs, specialized internet sites, and social media, among others, are becoming new database substitutes [24,25].

To our knowledge, there are no previous studies analyzing user-generated content from social media regarding nurses’ experiences. The current study uses the social media platform Instagram and emic material as a window to process the assessment of nurses’ experiences during the COVID-19 pandemic. By incorporating the nurses’ reviews shared on Instagram, we attempted to ascertain the dominant concepts used by nurses when reporting their experiences during the COVID-19 outbreak.

The current study contributes to nursing literature at least in three ways: first, we crystalize the main themes during the outbreak of COVID-19 as expressed by nurses on the social media platform Instagram. Second, we explore the inter-relationships among concepts expressed within Instagram captions shared by nurses during coronavirus. Third, we suggest important managerial insights for hospital administrations. Therefore, the aim of this paper was to determine the prominent themes shared on Instagram by nurses during the COVID-19 pandemic.

## 2. Methodology

### 2.1. Ethical Statement

**Figure 1:**
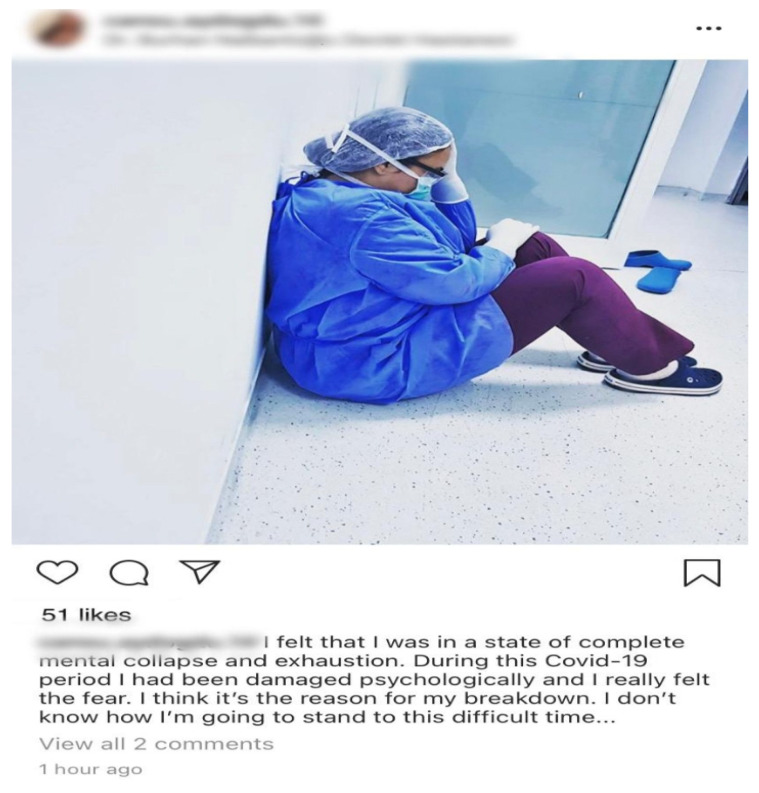
An example of a user caption extracted from Instagram used in the research.

All subjects gave their informed consent for inclusion before they participated in the study. The study was conducted in accordance with the Declaration of Helsinki, and the protocol was approved by the Ethics Committee of Cyprus West University (project identification code 2020/01–22). The Ethics Committee of Cyprus West University decided that the current research did not meet the definition of human subject research. This was on the basis that the current research was a review of publicly available social media posts and did not involve interactions with human subjects. All social media posts utilized in this research were publicly available and viewable to any website visitor [26,27]. Within the scope of gathering data, an Instagram caption of a nurse (Figure 1) was obtained, which was posted through an Instagram account. Due to ethical issues, the permission and consent of the mentioned nurse was requested through email. The nurse, whose identity will remain anonymous and whose confidentiality will be respected, voluntarily accepted to take part in the study with confirmation in response to our email.

### 2.2. Data Collection

The source for the captions analyzed in the present study was Instagram, which is considered one of the world’s largest social media platforms [28]. The Instagram social media platform offers a unique way for its users to share user-generated content, such as photos or videos, via their mobile phones. In 2019, there were almost 855 million users who accessed the photo-sharing platform Instagram every month. In 2023, this figure is projected to surpass 988 million users. Instagram is one of the most popular social media platforms worldwide [29]. 

Social media has become ubiquitous in modern life and provides a large and readily available platform through which the public may receive information about nursing [30]. One way to enhance the public’s knowledge about nursing is through the use of social media [31,32]. The Instagram accounts of nurses was the data source of this research. In the study, nurses’ private profiles were not taken into consideration, and only public Instagram accounts were selected. The present study’s primary data were the “texts” used as a caption in their photo-posting, written in English to avoid the need to translate the texts [33].

In this research, two of our specialist authors were included in the data collection process. The data collection and analysis respected the reviewers’ anonymity [34]. Photographs were a subject of focus in our study only to prove that the participants were nurses. Convenience sampling was used to ensure the amount of content needed to utilize the Leximancer software [35] and match the sample size of previous related research. For example, Pearce and Wu [36] analyzed 167 reviews, Rodrigues and colleagues used 603 reviews [34], and Tseng et al. [37] gathered 630 reviews.

In order to include eligible nurses in the dataset of the study, the nurses had to meet a number of inclusion criteria: (1) being a nurse and having posted a photo with scrubs; (2) posts should include a caption; (3) captions should be written in English. During searching for nurses’ profiles, some key hashtags were used to reach a high volume of nurses. The most popular hashtags were entered, including #nurse, #nursing, #nurselife, #nursepractitioner, #instanursing, #nurseproblems, #nursesrock, and #nurselife. The selected hashtags were those most-used among nurses according to recent data [38].

The data collected from Instagram were the nurses’ sex, residency, tags they used, and caption text. Data collection period started from May to June. According to the data collected, 444 Instagram captions were written by female nurses (88.8%) and 56 by male nurses (11.2%). Around a third or 32% of the captions were posted by European nurses, 56.4% by nurses from the United States, 4.2% by United Kingdom nurses, and 7.4% from other regions.

### 2.3. Data Treatment

Content analysis has earned popularity as an essential technique to understand the fast-growing form of communication on the web [35]. Content analysis is another commonly used approach in the data analysis of nursing research [39]. In health care, a qualitative approach aims to discover the multifaceted phenomena faced by nurses [40]. The idea and the basic ideologies of the methodologies, research aims and questions, and design and data collecting principles offer differences across qualitative and quantitative methodologies [39]. Content analysis is a broad term for a number of various approaches utilized to analyze text [41]. It is an organized coding and grouping method used for discovering large amounts of word-based data unobtrusively to determine patterns of the phrase used, their frequency, associations, and the structures of communication [42].

Content analysis can, thus, be achieved with structured quantitative or unstructured qualitative approaches [43]. These include calculating words and measuring visible text appearances or making sense of what is transcribed and extrapolating hidden insights from the data [35]. Content analysis is appropriate for answering questions, such as: what are the concerns of individuals regarding an incident? What reasons do individuals have to utilize or not utilize a service or method? [39]. Content analysis is well-suited to analyze the multifaceted, critical, and sensitive phenomena of nursing [39,44,45], such as experiences of the coronavirus pandemic. The current research was performed through content analyses of nurses’ Instagram captions using Leximancer, which converts word-based data from common language into semantic forms [33,41]. Unlike NVivo or ATLAS.ti, Leximancer 4.5 does not apply word frequency, or coding of terms and phrases [37,46]. Leximancer and its algorithms are utilized for investigating the senses within texts by mining the main concepts and concepts [35]. 

**Figure 2:**
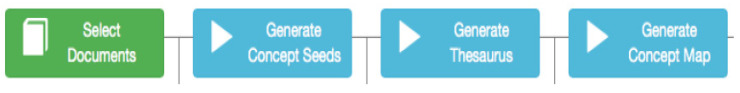
Project stages of the Leximancer software.

In the current study, there were four steps to determine the concept map as illustrated in Figure 4 below. To be able to generate the conceptual map, a number of steps were performed. First, the data we collected from Instagram were copied to an Excel file. Then, we added the Excel file into the software. As can be seen in Figure 2, we generated concept seeds as a second step. Concept seeds represent the starting point for the definition of concepts. They are single words that are the potential central keywords of distinct concepts [47]. The following phase was the thesaurus generation part. The thesaurus of terms was associated with each seed.

As mentioned earlier, concepts are collections of correlated words that encompass a central theme. Once these lists of words were identified for each concept, the concept map was generated to illustrate the relationships between the concepts in the text. The thesaurus displays a list of concepts, the number of iterations performed by the thesaurus learning system, while generalizing from the seed words to the concepts, and producing a ranked list of the thesaurus words that define and describe each concept [48]. The thesaurus list also shows the relevancy weightings associated with each indicative word. After these processes, a concept map, as illustrated in Figure 4, was generated.

**Figure 3:**
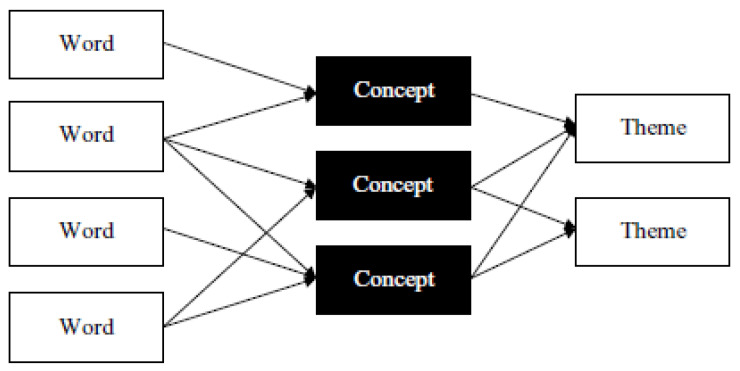
The basic model of the semantic configuration extraction in Leximancer. Source: adopted from Crofts and Bisman (2010).

Figure 3 illustrates the semantic pattern extraction in Leximancer. This shows the links among words, concepts, and themes in the software concept maps, which will be considered in the result interpretation section. Leximancer groups concepts into themes according to how often they appear together in a text [49].

As in earlier research utilizing Leximancer [35,37,50,51], these analyses were tracked with a narrative (i.e., qualitative) analysis, which acknowledged the texts, including the various themes. The research outcomes were interpreted according to the current conceptual frameworks of nurses’ experiences during coronavirus.

## 3. Results

**Figure 4:**
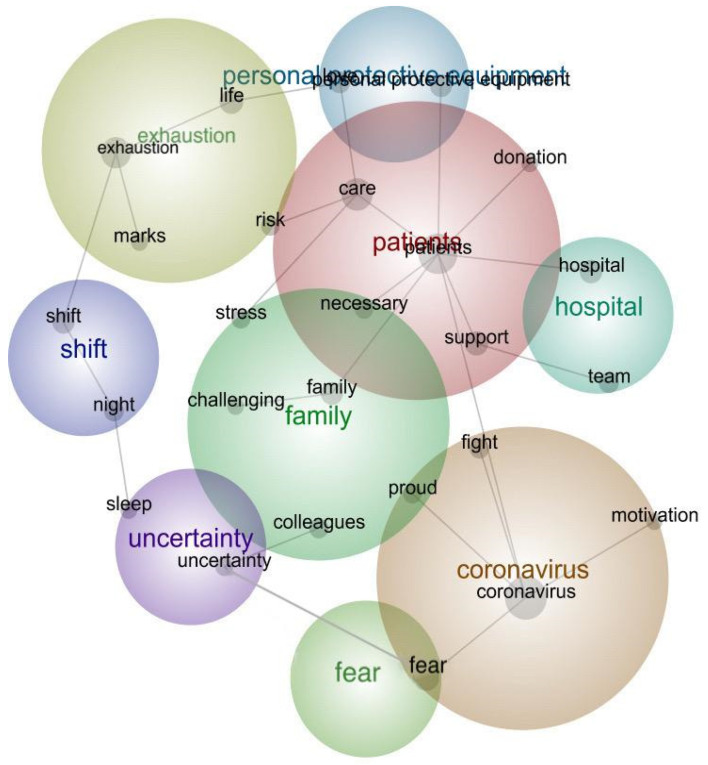
Concept map.

The content analyses revealed the existence of nine themes in the nurses’ descriptions of their experiences during the outbreak of COVID-19 (Figure 4): “patients”, “coronavirus”, “exhaustion”, “family”, “hospital”, “personal protective equipment”, “fear”, “shift”, and “uncertainty”. Table 1 shows the abovementioned themes and concepts and their relevant percentages.

As seen in Figure 4, nine central themes were found; almost all had a connectivity rate higher than 10%. The connectivity rate refers to the theme’s internal items stated closely with a particular percentage [36]. It shows the relative significance of themes (a theme with highest relevancy rate would be 100%).

We collected 500 Instagram captions shared by nurses (reviews) from the social media platform, Instagram. The dataset included 50,992 words in total. Figure 4 illustrates the concept map that was the basis for exploring the essence of the text. The nine main themes and their connectivity rates (in parentheses) were patient (100%), coronavirus (81%), exhaustion (76%), family (58%), hospital (51%), personal protective equipment (38%), fear (28%), shift (14%), and uncertainty (11%), in order of relative significance. The mentioned themes were the key themes shared through nurses’ Instagram posts, demonstrating their experiences during the COVID-19 outbreak. The relevancy rate obtained from the Leximancer software [48] considered the link of concepts within the themes and mirrored the significance of each theme.

The nine themes were found to be the essential aspects and the most-mentioned themes among nurses who worked during the pandemic. Figure 4 illustrates the themes and their linked concepts. For the software output, the slighter gray nodes are the concepts, and these are clustered with various rainbow-colored themes [37]. The most significant theme (patient) in this study was assigned the color red. Then, in descending order of significance, the other themes are represented by orange, yellow, green, blue, and purple.

Twenty-five concepts were recognized as a result of the analysis. The more the concepts were located within a theme, the more the sense of the theme was articulated. A preliminary understanding of Figure 4 may propose that it mirrors the mostly mentioned concepts and themes during the coronavirus experiences among nurses. The themes with more solid links to several concepts were patients, coronavirus, exhaustion, family, and hospital. By contrast, personal protective equipment, nurses, shift, and uncertainty had few linked concepts.

In this study, nurses shared narratives in their captions regarding some of the difficulties they faced during the pandemic. Long working hours, a lack of protective equipment, daily increased exhaustion, personal health, and the risk of loved ones facing outbreaks are just some of them. Looking at it historically, nurses have always been at the forefront of combating health-threatening outbreaks worldwide, and in the future, they will also continue to do so. This research was especially focused on nurses’ experiences during the coronavirus period.

In contrast to previous research, this study stressed the nurses’ natural language use in describing their experiences during the outbreak in their hospitals. Instagram captions were used as research materials. Leximancer 4.5 was utilized to analyze nurses’ Instagram captions during the coronavirus. The current research sought to accomplish three research objectives, the first of which was to identify the main themes in the descriptions of nurses’ experiences shared via the social media platform Instagram and then to find the relationship among concepts.

The study also analyzed the main components of the nurses’ experiences during a pandemic. Leximancer 4.5 was applied and identified nine major textual themes and the relationships among these themes. In order of relative importance, the themes were “patients”, “coronavirus”, “exhaustion”, “family”, “hospital”, “personal protective equipment” (PPE), “shift”, “fear”, and “uncertainty”.

The use of Leximancer delivered a more inclusive framework of the nurses’ experiences during the coronavirus outbreak by providing a visual concept map with philological conceptions. These approaches are different from other content analyses, which simply present word occurrences and links. The results from Leximancer noticeably demonstrate the examination of blog texts and make it stress-free to trace back to the essential lexical concepts.

## 4. Discussion

The purpose of this study was to investigate nurses’ experiences through analysing the main themes shared on Instagram by nurses during the COVID-19 pandemic. In the current research, patient and care concepts had a high likelihood percentage, which shows that although nurses were under challenging conditions, they paid great attention to patient care, which is their first and foremost duty. In their Instagram captions, nurses shared positive narratives regarding patient care. Consistent with our findings, one study found that nurses during their interviews emphasized their patients’ goodwill, respect, active cooperation, and gratitude during the COVID-19 pandemic [52].

Although nurses as a health ambassadors have to deliver health services for patients consistent with their major duties and ethical values [53], there was a point where a number of healthcare workers showed no inclination to care for patients in Taiwan and Canada during the SARS outbreak [54]. One of the concepts that the nurses mostly stressed in their Instagram caption was also “risk”. Nurses sometimes hold negative attitudes towards patients infected with infectious illnesses [55] as providing care for such patients may put health healthcare workers at risk of acquiring the infection [56].

Positive attitudes towards patients infected with transmissible diseases represent an essential element in the appropriate care of patients [57]. Nurses might experience an ethical paradox in deciding whether to provide treatment and care for patients with transmissible diseases. For example, unwillingness to treat patients with a transferrable virus was reported in 23% to 50% of healthcare workers in the US, 21% in Spain, and 14% in Canada [58].

In addition, one study demonstrated that nurses facing virus catastrophes displayed a clash between their roles as healthcare workers and feeling self-sacrificing, professionally accountable, fearful, and guilty regarding possibly transmitting the virus to their wife/husband, mother/father, or daughter/son at the same time [59]. Another study reported that the healthcare workers’ willingness to care for patients during the virus was their professional responsibility in nursing [60]. One study found that the virus did not produce a change in employees’ willingness to care for SARS patients and accept critical duties [61].

The second striking theme in the study was “coronavirus”. Coronavirus themes included concepts, such as fight, motivation, and proud. In the narratives of nurses that were shared on Instagram, the nurses shared narratives around “proud”, where they reflected on the coronavirus pandemic and they revealed that they were proud of their work. Nurses mentioned the theme of “fight” in their narratives towards coronavirus, which shows their dedication to helping fight the pandemic. Nurses shared stories using the concept of “motivation” during the coronavirus phase.

According to Min and colleagues [62], nurses perform in multifaceted circumstances that embrace the highest experience and capability in displaying a patient-oriented attitude and providing nursing care. The challenging work atmosphere in the hospital places a great deal of pressure on nurses’ shoulders. The advent of globalization has also injected a new phenomenon where a stressful work environment may diminish the nurses’ motivation levels. This scenario, if left unchecked, may hurt their job performance. In this study, nurses mentioned the concept of “motivation” with coronavirus themes according to the Leximancer software analysis.

In the study, the theme of exhaustion appeared as another main theme. The theme of exhaustion had a strong relationship with several concepts. Most of the exhaustion themes were linked to exhaustion, marks (due to PPE), shifts, and life. Exhaustion was a concept for nurses to express their thoughts and impressions during coronavirus in this current research study. Nurse exhaustion is a “work-related situation that ranges from severe to long-lasting in nature and can generate an overwhelming sense of tiredness, reduced dynamism, as well as tiredness resulting in impaired physical functioning, cognitive functioning, or both” [62].

The nurses caring for COVID-19 patients felt extreme physical exhaustion and pain triggered by the virus, heavy work circumstances, long shifts, a high volume of patients, and inadequate personal protective equipment, which was parallel with the past and present research on the outbreak of MERS-CoV [63] and Ebola [64]. A recent study demonstrated that COVID-19-associated stress is linked with exhaustion [65].

Another study investigating the link between burnout, anxiety, and stress disorders during the COVID-19 pandemic showed that nurses experienced high levels of mental health problems, including emotional exhaustion [66]. A qualitative research study performed during the COVID-19 pandemic showed that every nurse suffered overwhelming exhaustion [67]. A study conducted by Fiksenbaum and colleagues [68] revealed that the working conditions contributed considerably to an amplified perceived SARS threat, which caused increased exhaustion and anger.

In our study, the theme of “family” was used in different senses in the narratives shared by nurses on their Instagram accounts. In their narratives, some nurses called on people to stay home in order to prevent endangering their families with the possibility of coronavirus infection. Some nurses shared narratives of having coronavirus and transmitting the virus to their loved ones and families. The family theme was linked with concepts, such as family (20%), patients (18%), challenging (18%), and risk (14%).

Nurses, in general, face challenges in balancing work and family [61]. Research has shown that family is one of the most important factors behind nurses quitting their jobs [61,69]. One study confirmed that family is one of the factors responsible for a worker’s choice to stay [70]. Many nurses experienced social stigmatization (49%) and exclusion by family members (31%) during the time of SARS [71]. The strain and the struggle radically appear between work and family for healthcare workers when a catastrophe occurs. A study of the SARS in Canada showed that health care workers not only feared becoming infected themselves but also feared infecting family members, friends, and colleagues [72].

In our study, “uncertainty” was another mentioned theme. In line with our research, uncertainty was observed during the SARS period on nurses [73]. One study reported that several nurses who had been assigned to care for diseased patients in the event of avian flu said that hospitals’ support mechanisms did not appear to be adequate to meet this problem. The study also mentioned that the deficiency of understanding and uncertainty caused noteworthy dissimilarity in the establishment of information, which led to confusion and nervousness, which led to fear and panic among Taiwanese nurses. In agreement with our findings, one study reported that, during the outbreak of SARS, nurses reported uncertainty, information mismanagement, and feelings of anger, guilt, and unpreparedness [74].

Another theme that appeared in this study was “fear”, which shows the nurses shared narratives that included the theme of “fear” towards coronavirus. Nurses’ fears regarding an unknown infectious disease could quickly be intensified to higher levels. Although nurses make a promise to practice professionally, they can still feel fear and even experience worries regarding contagious diseases [75]. The results of our analysis display that nurses encountered a great deal of fear and anxiety when taking care of patients with coronavirus.

Consistent with our results, research has displayed that nurses often experience psychological problems, for instance depression, nervousness, sleeplessness, and strain, and those at the frontline of combating coronavirus have more severe psychological problems [76,77]. A recent study has shown that an elevated level of fear of COVID-19 was linked with lessened job satisfaction, increased psychological distress, and increased turnover intentions [78]. Shanafelt and colleagues (2020) identified the sources of fear among nurses, including lack of personal protective equipment (PPE) and fear of transmitting the virus at work [79]. Using a phenomenological approach, twenty participants were included in the study and asked about their COVID-19 experiences. All interviewees expressed their fears, which peaked when they entered the negative pressure ward in the first wave of the outbreak [52].

The analyses of this research reveal that personal protective equipment (PPE) is the critical issue that nurses share narratives on. Concepts, such as “necessary” and “marks” (left on their faces), were used together with PPE in the narrative of nurses. The perceived absence of PPE was a factor in nurses’ distress and worries regarding working during pandemics [80]. Protecting nurses is essential during the COVID-19 period and requires personal protective equipment (PPE).

In addition to a deficiency of PPE causing fear and distress among nurses, skin reactions from the gloves, gowns, or face shields that were also worn by nurses for long hours during the existing pandemic discouraged nurses from using them [81]. The wearing of PPE exponentially augmented dermatological problems, which was common among nurses [82]. In line with this information, a study undertaken in China has demonstrated an occurrence of 97% of skin damage related to PPE among health workers during the COVID-19 pandemic [83]. The exposure period is the prevalent risk factor for face dermatitis—primarily, wearing masks for over 6 h [84].

## 5. Conclusions

The aim of this study was to find the main themes shared on Instagram by nurses during the COVID-19 pandemic. Leximancer 4.5 was applied and used to identify nine major textual themes and the association among these themes. In order of the relative importance, the themes were “patients”, “coronavirus”, “exhaustion”, “family”, “hospital”, “personal protective equipment” (PPE), “shift”, “fear”, and “uncertainty”. The use of Leximancer portrayed a more inclusive framework of the nurses’ experiences during the coronavirus outbreak by providing a visual concept map. These approaches are different from other content analyses that simply present the word occurrences.

Findings from this research show that the coronavirus substantially affected the psychological state, workload, health provisions, and safety concerns of nurses. The mentioned factors also have the potential to impact the quality of care given by nurses. This study provides nurse leaders and policymakers with nurse-oriented evidence to comprehensively plan and better optimize the allocation of nursing resources in line with the aims and objectives of healthcare centers during the COVID-19 pandemic.

### 5.1. Theoretical Contributions

This research offers insights into understanding the perception of nurses during the coronavirus pandemic, based on the narratives these nurses shared online. Leximancer’s analytical tools enabled the identification of the main themes in nurses’ overall experiences during a pandemic with minimal intervention from the researchers. There have been a few studies that have analyzed the COVID-19 experiences of nurses using quantitative methods [19]. However, our research focused on narratives shared by nurses through social media platforms, which is vital as none of the previous studies have considered the nurses’ experiences using a content analysis approach.

### 5.2. Practical Implications

Nurses’ sense of duty, commitment to patient care, personal sacrifice, and professional teamwork increased during the COVID-19 pandemic. Although nurses shared narratives on fears, family welfare, and panic, as well as defenselessness, their positive narratives and hashtags also demonstrated care for their patients and how much they love their job. Almost all nurses shared narratives supporting a willingness to accept the jeopardies of their duty during the COVID-19 pandemic. The important effect of nurses’ experiences highlights a need for strategies around self-care and ongoing support to ensure that the health of nurses is maintained.

Hence, a method of increasing the quality of patient care might be an attempt to set or improve the best-fit bundle of human resource practices and provide sufficient resources in the implementation of high performance work practices (HPWP). Recent studies have shown that HPWS improved patient care quality among nurses [85,86]. High-performance work practices were also proposed to be performance boosting as human resource systems can assist management to improve the work engagement of employees [87]. Hospital superiors should also concentrate on how to appraise nurses’ performance and to develop teamwork to be able to improve motivation during the COVID-19 pandemic.

Work related fatigue includes not only physiological aspects but also emotional, cognitive, and sensory elements that result from a high workload and insufficient time for energy recovery [88]. In the current study, “exhaustion” and “shift” were key concepts, which signifies that nurses had difficulties during their shift both mentally and physically. Several steps can be taken in this regard. There was a consensus among several scholars that the psychological aspects of work affect fatigue and that there is a necessity for rescue among nurses working 12 h shifts. A mounting body of evidence agrees that nurse exhaustion is a pivotal reason for nursing staff deficiencies and adverse patient consequences [89,90].

To minimize nurse exhaustion during the coronavirus period and the associated adverse nurse and patient consequences, additional useful or achievable characteristics of work structures should be reformed (e.g., more beneficial scheduling to permit good quality sleep and encourage help for these initiatives by administrators as well as associates). Hospitals might reflect shorter working hours, systematic rest periods, and rotating shifts for those working in high-risk areas if possible [91]. A novel review study has stated that generating a helpful work climate in hospitals, particularly by nurse leaders, can help to avoid nurse exhaustion and tension [92].

In the study, the narratives shared by nurses repeated the theme of “personal protective equipment” (PPE). The results of the analysis show that the PPE theme was associated with the concept of “necessary” as well as “risk”. Undoubtedly, the necessity of PPE created a problem of immense distress and intensified the risk and anxiety for nurses. Therefore, PPE should be utilized according to the risk of experience (e.g., type of activity) and the contamination dynamics of the pathogen (e.g., contact, droplet, or aerosol). The excessive utilization of PPE will have an extra effect on stock deficiencies. Therefore, the “rational use of personal protective equipment (PPE) for COVID-19” prepared by World Health Organization [93] should be followed to avoid PPE deficiencies. Following these recommendations, an organization will ensure the balanced use of PPE. As a result of this, nurses will lessen their anxiety regarding transmission possibilities due to inadequate PPE.

Another theme that appeared in this study was “uncertainty”. Previous research has stated that uncertainty occurs in a situation where an event or a disease cannot be adequately defined or categorized, or the outcome predicted [45]. In the phenomenon of caring for coronavirus patients, uncertainty is present during the treatment. These circumstances are multifaceted and provided only irregular, unaware, inconsistent, and limited information on how to treat and cope with this novel virus. Therefore, hospitals should have held more training on how to treat coronavirus patients. Most of the nurses mentioned the theme of “fear” in their narratives. Over time, in-service training might also diminish nurses’ fears regarding the potential results of caring for patients with coronavirus.

### 5.3. Limitations and Avenues for New Research

The current research cannot be generalized as it has certain limitations. The first limitation is the sample that was used in our research. We collected our data from Instagram. It would be interesting to conduct this research with more participants. The second limitation of this research is the cross-sectional time span used in this study. Due to this, there is potential causality and reciprocal associations between components [94]. This study was exploratory to produce a better understanding of the nurses’ experiences during the coronavirus battle. More quantitative and qualitative research are undoubtedly required to further clarify nurses’ experiences towards coronavirus.

Our study collected data from a single social media platform—Instagram. Further studies could compare the user-generated content of nurses from different social media platforms (e.g., Facebook and Twitter) to analyze the relevance in comparison with this paper’s findings. Different research methods will be useful, for instance, surveys and interviews with nurses who have encountered coronavirus. These further studies may focus on the intention to care, COVID-19 patients, psychological distress among nurses, and uncertainty and its effects on nurses. Future research may expand the range of available data and provide comparisons among different countries-of-origin and different cultural groups to expand the knowledge regarding nurses’ experiences during the pandemic.

## Figures and Tables

**Table 1:** Main themes, concepts, and relevancy percentages.

Themes	Concepts	Relevancy
Patients	patient	100%
	care	44%
	risk	41%
	support	33%
	donation	27%
	necessary	38%
Coronavirus	coronavirus	37%
	motivation	31%
	proud	28%
	fight	19%
Exhaustion	exhaustion	39%
	marks	30%
	life	21%
Family	family	20%
	patients	18%
	challenging	18%
	risk	14%
Hospital	hospital	18%
	team	11%
Shift	shift	15%
	night	9%
Uncertainty	uncertainty	11%
	sleep	9%
Fear	fear	12%
Personal protective equipment	Personal protective equipment	12%
